# Autoimmune disease mouse model exhibits pulmonary arterial hypertension

**DOI:** 10.1371/journal.pone.0184990

**Published:** 2017-09-19

**Authors:** Koichi Sugimoto, Kazuhiko Nakazato, Akihiko Sato, Satoshi Suzuki, Akiomi Yoshihisa, Takeshi Machida, Shu-ichi Saitoh, Hideharu Sekine, Yasuchika Takeishi

**Affiliations:** 1 Department of Cardiovascular Medicine, Fukushima Medical University, Fukushima, Japan; 2 Department of Pulmonary Hypertension, Fukushima Medical University, Fukushima, Japan; 3 Department of Immunology, Fukushima Medical University, Fukushima, Japan; Universite Paris-Sud, FRANCE

## Abstract

**Background:**

Pulmonary arterial hypertension is often associated with connective tissue disease. Although there are some animal models of pulmonary hypertension, an autoimmune disease-based model has not yet been reported. MRL/lpr mice, which have hypergammaglobulinemia, produce various autoimmune antibodies, and develop vasculitis and nephritis spontaneously. However, little is known about pulmonary circulation in these mice. In the present study, we examined the pulmonary arterial pressure in MRL/lpr mice.

**Methods and results:**

We used female MRL/lpr mice aged between 12 and 14 weeks. Fluorescent immunostaining showed that there was no deposition of immunoglobulin or C3 in the lung tissue of the MRL/lpr mice. Elevation of interferon-γ and interleukin-6 was recognized in the lung tissue of the MRL/lpr mice. Right ventricular systolic pressure, Fulton index and the ratio of right ventricular weight to body weight in the MRL/lpr mice were significantly higher than those in wild type mice with same background (C57BL/6). The medial smooth muscle area and the proportion of muscularized vessels in the lung tissue of the MRL/lpr mice were larger than those of the C57BL/6 mice. Western blot analysis demonstrated markedly elevated levels of prepro-endothelin-1 and survivin as well as decreased endothelial nitric oxide synthase phosphorylation in the lung tissue of the MRL/lpr mice. Terminal deoxynucleotidyl-transferase-mediated dUTP nick end-labeling assay showed the resistance against apoptosis of pulmonary arterial smooth muscle cells in the MRL/lpr mice.

**Conclusion:**

We showed that MRL/lpr mice were complicated with pulmonary hypertension. MRL/lpr mice appeared to be a useful model for studying the mechanism of pulmonary hypertension associated with connective tissue diseases.

## Introduction

Pulmonary hypertension often complicates connective tissue disease (CTD) and determines its prognosis. Recently, the survival of patients with CTD-associated pulmonary hypertension (CTD-PH) has been improved by using targeted pulmonary vasodilators or active immunosuppressive therapy [1]. However, the outcome is still insufficient and the mechanism of CTD-PH remains unclear [2].

The characteristics of the pulmonary arteries in CTD-PH are supposed to be similar to those of idiopathic pulmonary arterial hypertension (IPAH), and they consist of vasoconstriction and organic lumen narrowing due to abnormal proliferation of endothelial or smooth muscle cells.

Immunologically, T lymphocytes differentiate into T helper (Th) 1, Th2, Th17, and regulatory T cells, and imbalance of Th1/Th2/Th17 and regulatory T cells contributes to the pathogenesis of CTD [3, 4]. In addition, interleukin (IL)-6 is known to be a key molecule in pulmonary arterial remodeling in pulmonary hypertension [5]. However, detailed mechanisms of CTD-PH have remained still unclarified.

According to the Nice classification, CTD-PH is classified into Group 1 (pulmonary arterial hypertension) as IPAH because the treatment methods are similar to those for IPAH [6]. However, CTD-PH also has characteristics of Group 1’ (pulmonary vein occlusion), Group 2 (pulmonary hypertension due to left sided heart disease), and Group 3 (pulmonary hypertension due to lung diseases) because it sometimes accompanies pulmonary vein occlusion, fibrosis of the left ventricular myocardium, and interstitial pneumonia. Further, CTD-PH, except in case of scleroderma, can be expected the improvement by immunosuppressive therapy [1, 7], which is another way in which CTD-PH differs from IPAH. Thus, to approach clinical CTD-PH, an experimental model of CTD that spontaneously develops pulmonary hypertension is necessary in addition to monocrotaline-administered mice and vascular endothelial growth factor (VEGF) inhibition with hypoxic exposure mice which are popular as animal models of pulmonary arterial hypertension [8, 9].

MRL/lpr mice spontaneously develop vasculitis and glomerulonephritis due to hypergammaglobulinemia and expression of various autoantibodies. They are widely used as models for lupus nephritis and Sjoegren's syndrome [10].

However, little is known about the onset of pulmonary hypertension in these mice.

In the current study, we examined the hemodynamics and histopathological features of pulmonary vessels, the expression of molecules associated with pulmonary vasoconstriction and vasodilatation, as well as medial smooth muscle cell apoptosis in MRL/lpr mice.

## Methods

### Animals and ethics statement

MRL/lpr mice (#000485) were purchased from Jackson lab (Bar Harbor, ME, USA). We used female MRL/lpr and C57BL/6 mice aged between 12 and 14 weeks (Body weight range was from 19.7 to 32.5 g). As positive controls for fluorescent immunostaining of C3 and immunoglobulin, kidneys of 23-week-old MRL/lpr mice were used. Mice were housed with food and water ad libitum at room temperature under a 12 h: 12 h light-dark cycle. The investigations conform to the Guidelines for the Care and Use of Laboratory Animals published by the US National Institutes of Health (NIH publication, 8^th^ Edition, 2011). Our research protocol was approved by the Fukushima Medical University Animal Research Committee. All efforts were made to minimize the suffering of the animals. All of the mice were sacrificed by cervical dislocation after the experiments.

### Measurements of right ventricular pressure and ventricular weight

Anesthesia was performed by intraperitoneal injection of Tribromoethanol (0.25 mg/g of body weight). A 1.2F micromanometer catheter (Transonic Scisense Inc., London, ON, Canada) was inserted from the right jugular vein, and right ventricular pressure was measured and analyzed by LabScribe3 software (IWORX, Dover, NH, USA). In order to evaluate right ventricular hypertrophy, right ventricle (RV) was dissected from the left ventricle (LV) including septum (S), and the RV/LV+S weight ratio and RV/body weight ratio were calculated [11, 12].

### Histological analysis

After measurement of RV pressure, the lungs were fixed with 4% paraformaldehyde, embedded in paraffin, and sectioned to 3 μm. After Elastica-Masson (EM) staining or immunostaining of α-smooth muscle actin (α-SMA) (Santa Cruz Biotechnology Inc., Santa Cruz, CA, USA), pulmonary arteries (external diameter of 20–50 μm) were randomly selected (60–90 vessels per individual mouse). The medial wall area (the area between the internal and external lamina) was measured by Image J 1.48 (National Institutes of Health, Bethesda, MD, USA) and was divided by the vessel area (the area surrounded by the external lamina) [12]. Each vessel (external diameter < 25 μm) was classified as non-muscular, partially muscular or fully muscular. The percentage of muscularized pulmonary vessels was determined by dividing the sum of partially and fully muscular vessels by the total number of vessels [8, 12]. Measurements were performed blinded to mouse information.

Deposition of immunoglobulin (IgG) and C3 in the lung tissue was stained using fluorescein isothiocyanate (FITC)-labeled primary antibody [10]. Briefly, frozen sections (8 μm) fixed with acetone were washed with phosphate buffered saline (PBS) for 5 minutes 3 times and blocked with PBS containing 3% bovine serum albumin (BSA) at room temperature for 1 hour. The sections were stained by FITC-conjugated goat anti-mouse C3 antibody (MP Biomedicals, Solon, OH, USA) or FITC-conjugated rat anti-mouse IgG antibody (BioLegend, San Diego, CA, USA) diluted 1:100 with PBS containing 1% BSA for 1 hour at room temperature. After washing 3 times with PBS and deionized water, fluorescent images were captured with a fluorescence microscope (BZ-X700, KEYENCE Co., Osaka, Japan) at fixed exposure times. We used kidney tissue from 23-week-old MRL/lpr mice as a positive control.

### Assessment of cytokines in lung tissue of MRL/lpr mice

Levels of cytokines in the lung tissue of the MRL/lpr mice were measured using mouse Th1/Th2/Th17 cytokine kit (BD Biosciences, San Jose, CA, USA). Detectable cytokines by this kit were Th1-related cytokines (IL-2, interferon (IFN) -γ, tumor necrosis factor (TNF)), Th2-related cytokines (IL-4, IL-6, IL-10) and Th17-related cytokine (IL-17A). The lung tissue samples were solubilized with lysis buffer (10 mM Tris, 2 mM EDTA, 20 μg/ml antipain, 20 μg/ml leupeptin, 1 μM DTT and 1 μM PMSF). The protein concentrations in the lysates were then measured using the Bradford method and adjusted to 3 mg/ml. Capture beads conjugated with antibodies specific for each cytokine were added to the lysate of the lung tissue. These samples were incubated with phycoerythrin-conjugated antibody for 2 hours at room temperature in the dark. After a sandwich complex was formed, fluorescent intensity was measured by flow cytometry (BD FACS Canto II, BD Bioscience) and analyzed by Flow Jo^™^ v10.3 Software.

### Western blotting

Western blot was performed as described previously [13, 14]. The lysates of lung tissues were mixed at a ratio of 4:1 with loading buffer (75 mM Tris-HCl, pH 6.8; 10% glycerol; 3% 2-mercaptoethanol, and 2% sodium dodecyl sulfate (SDS)) and heated at 95°C for 10 minutes. Aliquots containing 20 μg of protein were subjected to SDS-polyacrylamide gel electrophoresis, and the proteins were then transferred onto polyvinylidene difluoride membranes (MILLIPORE, Bedford, MA, USA). After incubation with blocking solution at room temperature for 30 minutes, the membranes were incubated for 1 hour at room temperature with a mouse monoclonal antibody to endothelial nitric oxide synthase (eNOS) (Transduction Laboratories, Lexington, KY, USA) diluted 1:1000, β-actin (Santa Cruz Biotechnology, Santa Cruz, CA, USA) diluted 1:1000, or a rabbit polyclonal antibody to phosphorylated-eNOS (Ser 1177, Cell Signaling Technology, Beverly, MA, USA) diluted 1:1000, and survivin (Cell Signaling Technology) diluted 1:500. We used a rabbit polyclonal antibody against an epitope containing amino acid 8–16 of endothelin-1 (ET-1) (Novus Biologicals USA, Littleton, CO, USA) diluted 1:500. Each membrane was then subsequently incubated for 45 minutes with a horseradish peroxidase-conjugated goat anti-mouse or anti-rabbit IgG antibody, diluted 1:10000 (Santa Cruz Biotechnology). The signals from immunoreactive bands were visualized with an Clarify^™^ Western ECL Substrate (Bio-Rad Laboratories, Inc, Hercules, CA, USA). The optical densities of individual bands were analyzed using Image J 1.48.

### Apoptosis of pulmonary smooth muscle cells

To estimate the apoptosis of pulmonary smooth muscle cells, we performed a terminal deoxynucleotidyl-transferase-mediated dUTP nick end-labeling (TUNEL) assay (Promega, Madison, WI, USA) according to the manufacture’s instruction. We randomly selected at least 10 fields in each specimen and counted nuclei in the medial smooth muscle layer. The result was expressed as a percentage of the number of TUNEL-positive nuclei in the total number of nuclei.

### Statistical analysis

Data are expressed as mean ± SD, and statistical analyses were performed using Mann-Whitney U test. A value of P < 0.05 was considered statistically significant.

## Results

### Deposition of C3 and immunoglobulin

**Fig 1:**
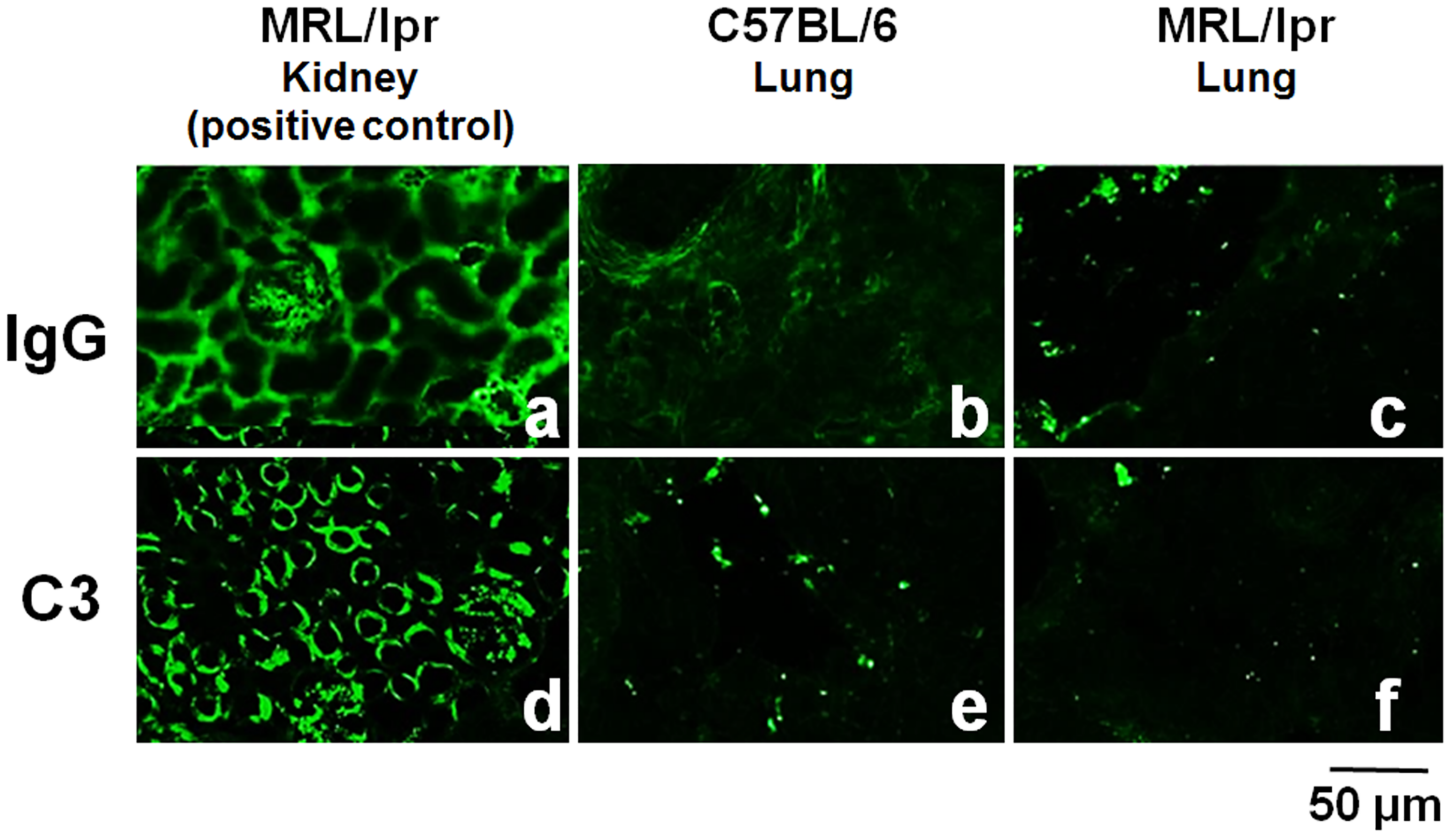
Deposition of C3 and IgG on the lung tissue of MRL/lpr mice. Immunofluorescent staining revealed that both C3 and IgG deposition are recognized in kidney tissue as positive controls (a, d), however, neither were detectable in the lung tissue of C57BL/6 (b, e) or MRL/lpr mice (c, f).

It has previously been reported that deposition of C3 and IgG is recognized on renal glomeruli in MRL/lpr mice [10], therefore, in the present study, we investigated whether similar findings were found in lung tissue. Fluorescent immunostaining revealed that deposition of C3 and IgG were not detected in the lung tissue (Fig 1b, 1c, 1e and 1f), whereas those were clearly visualized in renal glomeruli as positive controls (Fig 1a and 1d).

### Cytokines in lung tissue of MRL/lpr mice

**Fig 2:**
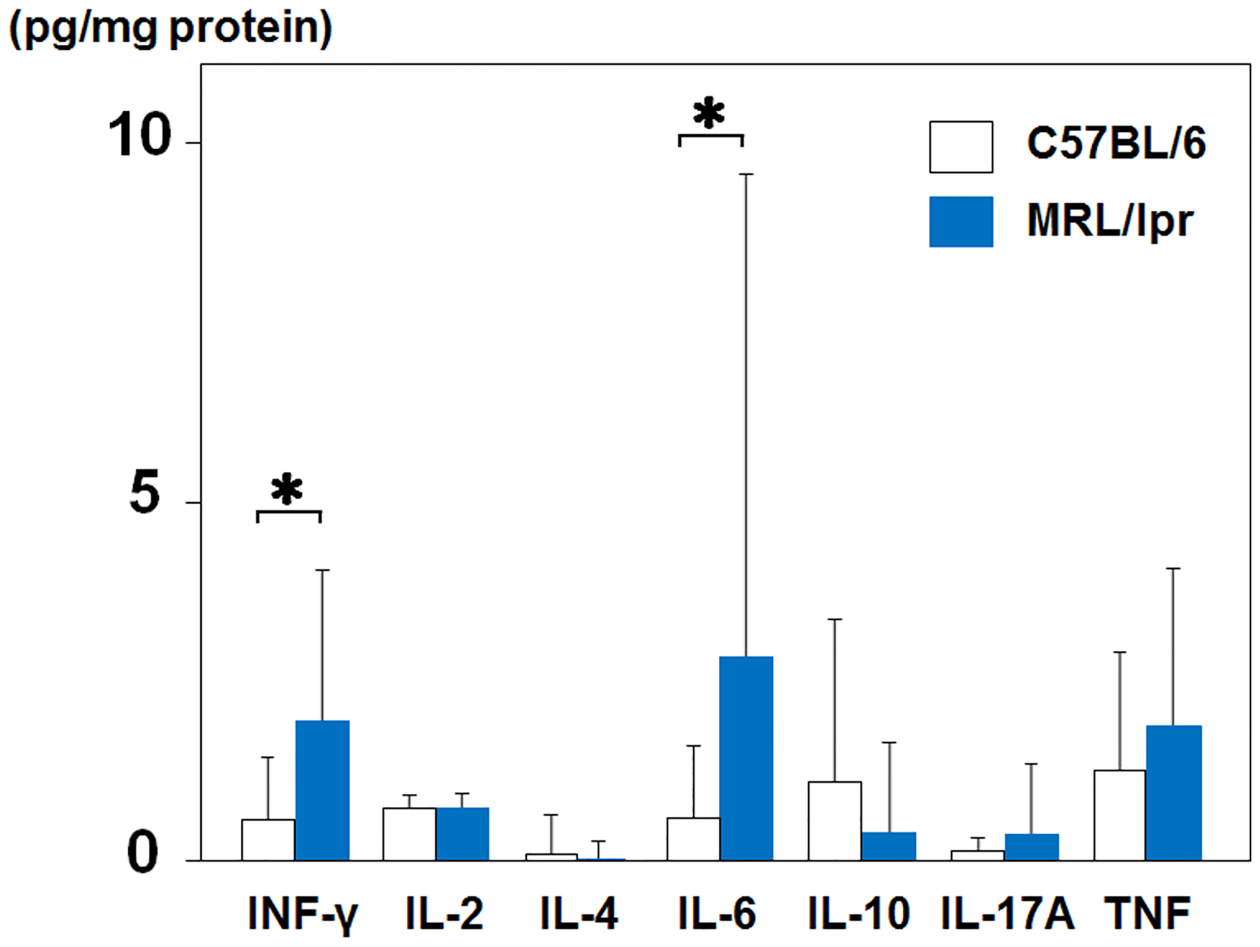
Cytokines in the lung tissue of MRL/lpr mice. The levels of cytokines in the lung tissue were assessed as described in the Methods. (open bar; C57BL/6 mice, solid bar; MRL/lpr mice). Results are expressed as mean ± S.D. of 9 to 11 animals. *P<0.05 vs. C57BL/6 mice.

Next, we assessed cytokine profiles of the C57BL/6 mice and the MRL/lpr mice. In the lung tissue of the MRL/lpr mice, the levels of IFN-γ and IL-6 were significantly higher compared with those of the C57BL/6 mice (2.0 ± 1.1 vs. 0.58 ± 0.44 pg/mg protein, P < 0.05, 2.9 ± 3.4 vs. 0.6 ± 0.51 pg/mg protein, P < 0.05). Although there was no statistical significance, IL-17A and TNF were higher whereas IL-10 was lower in the MRL/lpr mice. IL-4 in the lung tissue of the MRL/lpr mice was almost undetectable in this assay. IL-2 levels were nearly equivalent in the two groups (Fig 2).

### Right ventricular pressure and hypertrophy

**Fig 3:**
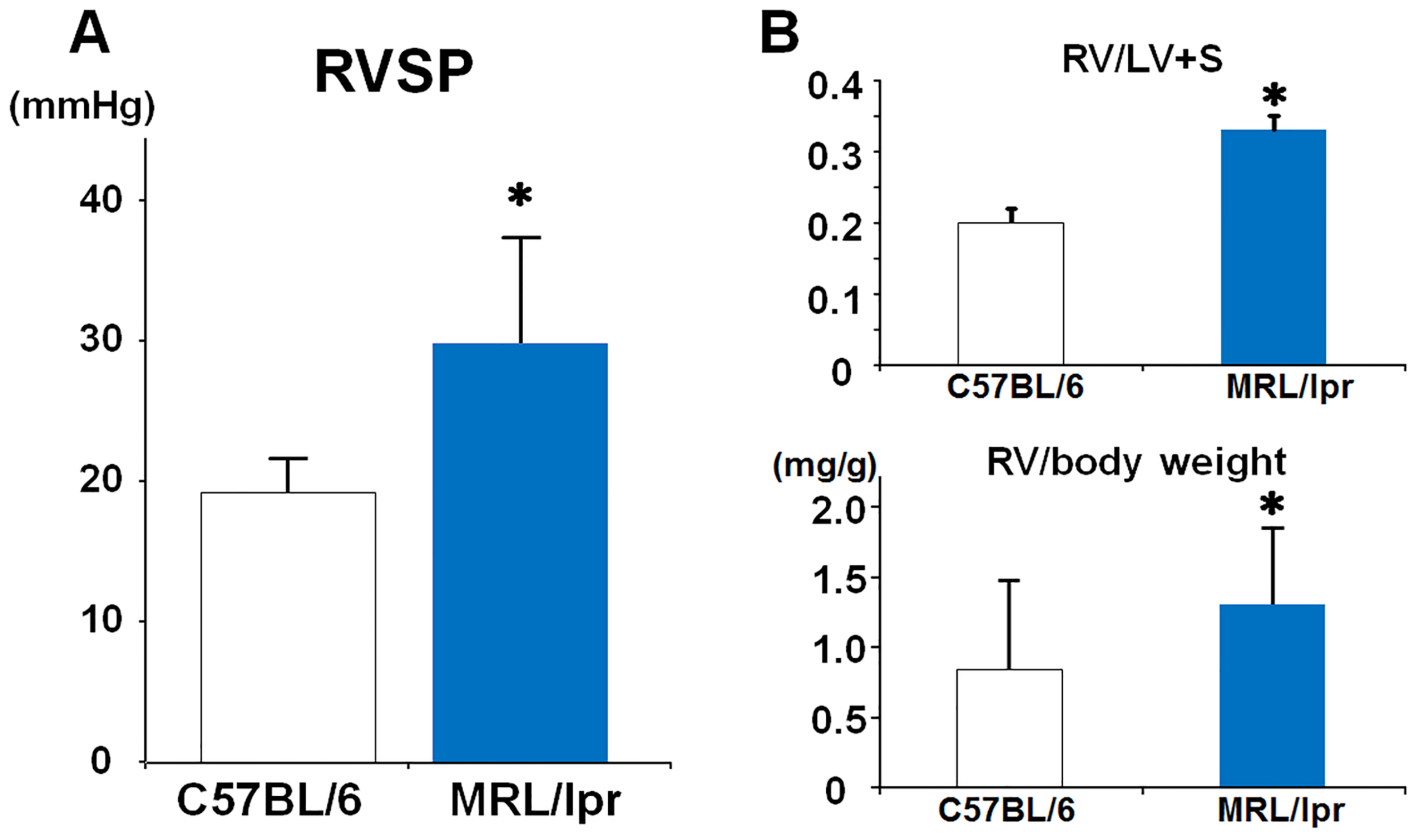
Measurement of RVSP and ventricular weight in MRL/lpr mice. RVSP in C57BL/6 and MRL/lpr mice (A). Results are expressed as mean ± S.D. of 5 to 6 animals. *P<0.05 vs. C57BL/6 mice. RV/LV+S and RV/body weight (B) in C57BL/6 and MRL/lpr mice. Results are expressed as mean ± S.D. of 8 animals. *P<0.05 vs. C57BL/6 mice.

There was a significant increase in right ventricular systolic pressure (RVSP) in the MRL/lpr mice (29.8 ± 7.2 vs. 19.2 ± 1.9 mmHg, P < 0.05) (Fig 3A). In this study, the penetration of PH in the female MRL/lpr mice was 83.3% (5/6). The RV/LV+S and RV/body weight were significantly larger in the MRL/lpr mice than in the C57BL/6 mice (0.33 ± 0.58 vs. 0.20 ± 0.67, P < 0.05, 1.3 ± 0.27 vs. 0.84 ± 0.31 mg/g, P < 0.05), (Fig 3B).

### Pulmonary medial wall thickening and vessel muscularization in MRL/lpr mice

**Fig 4:**
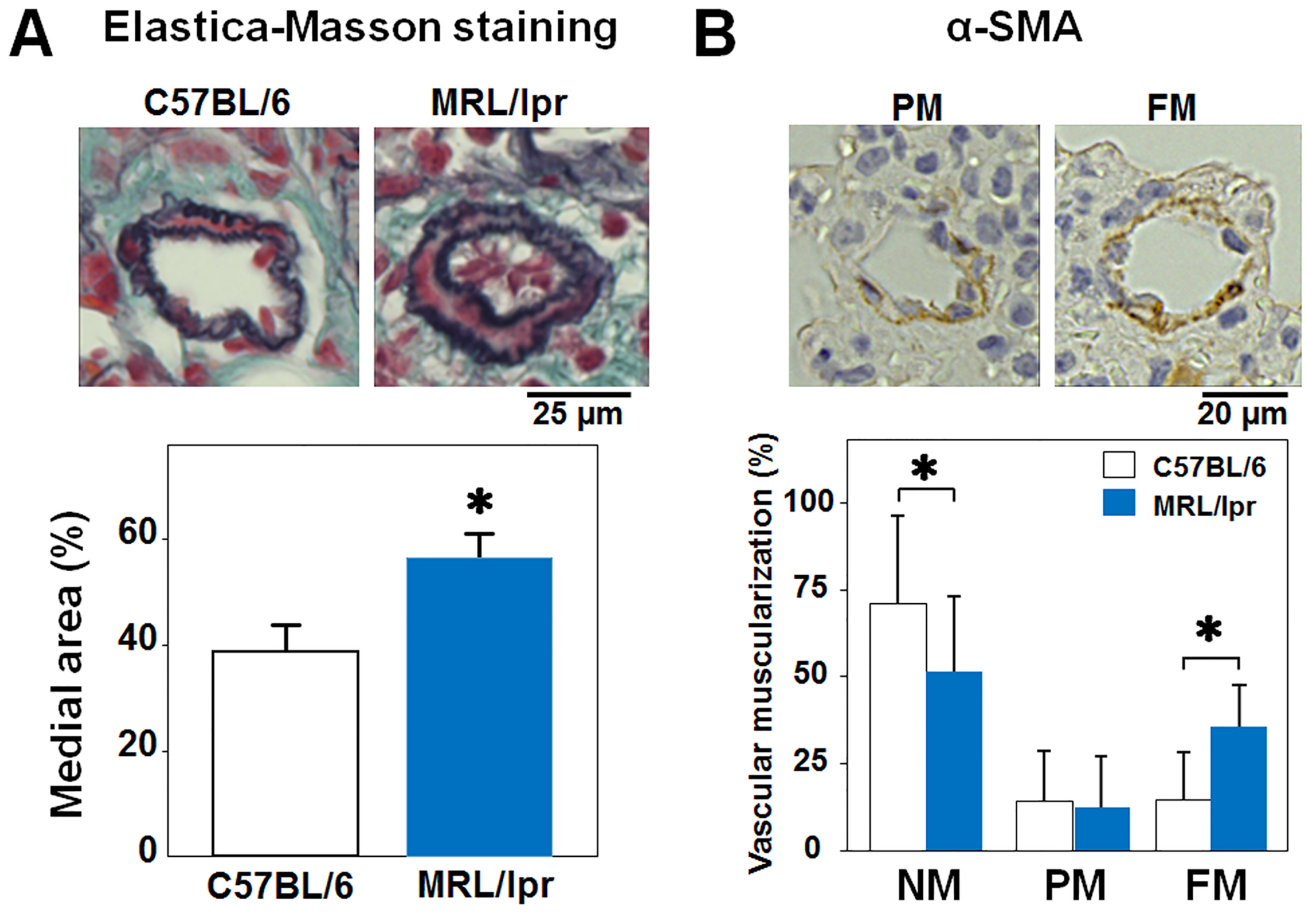
Medial wall thickening and muscularization of the pulmonary arteries in MRL/lpr mice. Representative microphotographs of the pulmonary arteries in C57BL/6 mice and MRL/lpr mice stained by EM staining (A) and α-SMA (B). A: Medial wall area was assessed as described in the Methods. B: Peripheral pulmonary arteries were classified into NM (non-muscular), PM (partially muscular), and FM (fully muscular) according to the degree of muscularization (open bar; C57BL/6 mice, solid bar; MRL/lpr mice). Results are expressed as mean ± S.D. of 5 animals. *P<0.05 vs. C57BL/6 mice.

Since the pulmonary medial wall thickening and muscularization of the peripheral pulmonary arteries are the major pathogenesis of pulmonary arterial hypertension [12, 15], we next observed the lung sections with EM staining and immunostaining of α-SMA. The medial smooth muscle layer of the MRL/lpr mice was significantly greater than that of the C57BL/6 mice (Fig 4A). In addition, the proportion of fully muscular vessels in the MRL/lpr mice was significantly higher than that in the C57BL/6 mice, whereas the proportion of non-muscular vessels in the MRL/lpr mice was lower (Fig 4B). The percentages of partially muscular vessels were nearly equivalent between the two groups.

### Vasodilation and vasoconstriction related molecules in lung tissue of MRL/lpr mice

**Fig 5:**
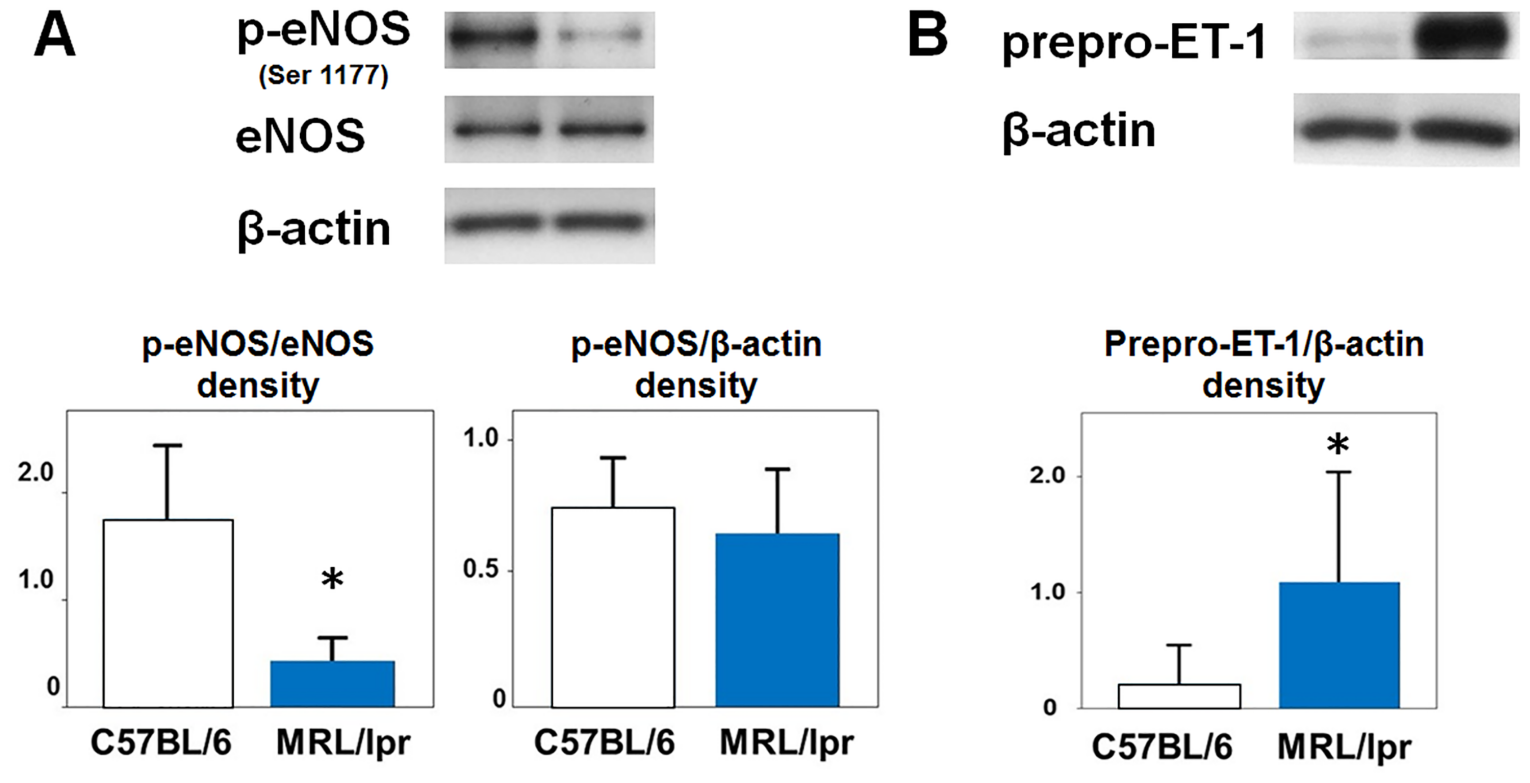
Decreased eNOS activation and increased prepro-ET-1 expression in the lung tissue of MRL/lpr mice. eNOS expression and phosphorylation (A), and prepro-ET-1 expression (B) were determined by western blotting. Representative immunoblots (upper panels) and graphs (bottom panels) are shown. Bars are mean ± S.D. of quantitative densitometric analyses; n = 5 for each experimental group; *P<0.05 vs. C57BL/6 mice.

Vasoconstriction is caused by the imbalance between vasoconstrictive factor and vasodilator; the former includes ET-1 as well as oxidative stress, and an example of the latter is nitric oxide (NO). The expression and activation of eNOS are important in the production of NO [16, 17]. Therefore, we investigated the expression and activation of eNOS in the lung tissues of the MRL/lpr mice by western blotting. The level of eNOS expression of the MRL/lpr mice did not differ significantly between the two groups; however, eNOS phosphorylation was significantly decreased in the MRL/lpr mice (Fig 5A). Western blot using a primary antibody against an ET-1 epitope demonstrated the bands around 28-kDa. Since a mature form of ET-1 has a small molecular weight (2.5-kDa), these 28-kDa bands were considered to show prepro-ET-1 as reported previously [18]. The expression of prepro-ET-1 in the lung tissue of the MRL/lpr mice was significantly elevated compared to that of the C57BL/6 mice (Fig 5B). These results suggest that vasoconstriction due to NO impairment and increased ET-1 production is one of the pathogenic mechanisms of pulmonary hypertension in MRL/lpr mice.

### Expression of survivin and apoptosis of pulmonary arterial smooth muscle cells in lung tissue of MRL/lpr mice

**Fig 6:**
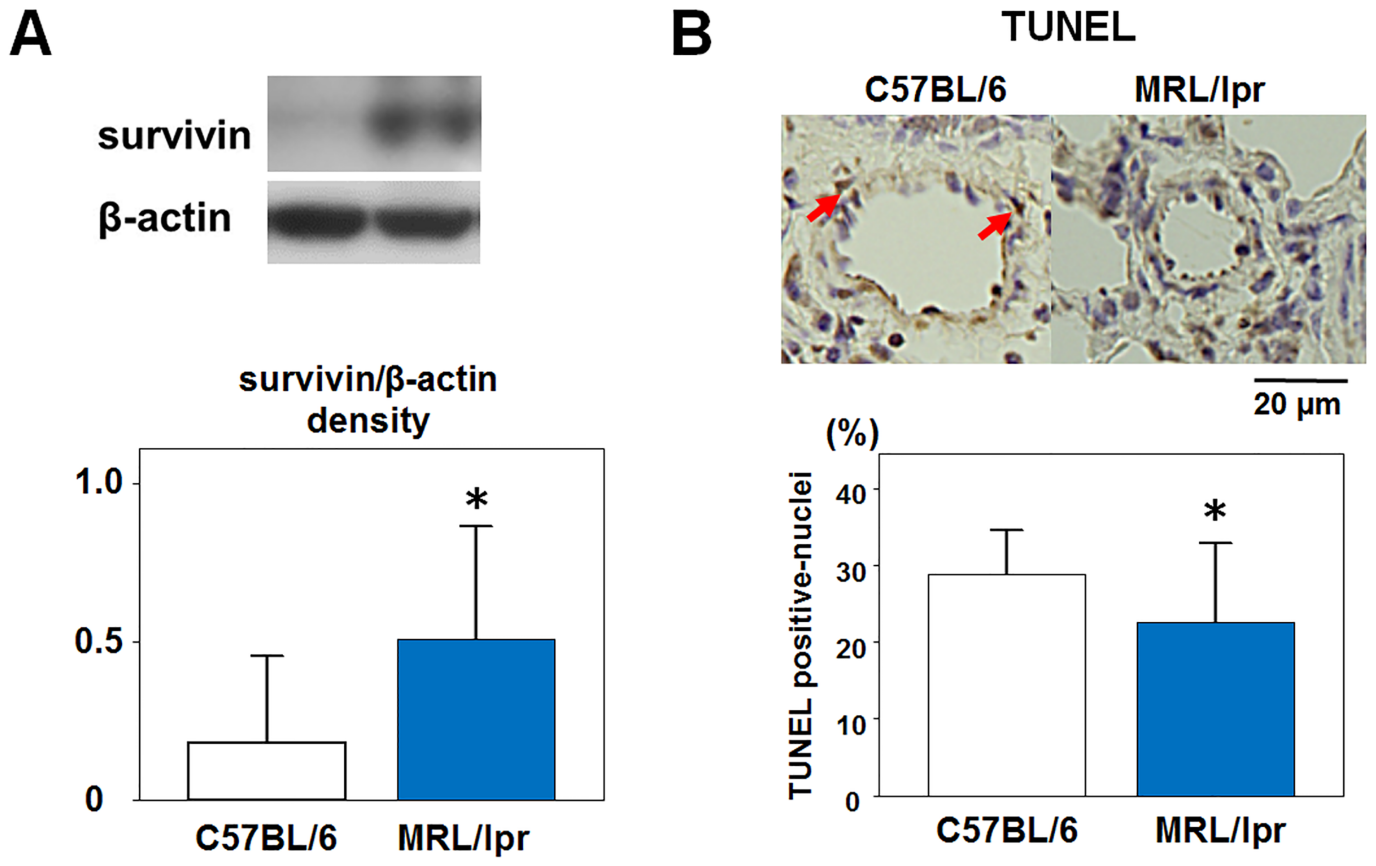
The levels of survivin expression and apoptosis of pulmonary arterial smooth muscle cells in the lung tissue of MRL/lpr mice. Survivin expression in the lung tissues was determined by western blotting (A). Representative immunoblots (upper panels) and graph (bottom panel) are shown. Bars are mean ± S.D. of quantitative densitometric analyses; n = 5 for each experimental group; *P<0.05 vs. C57BL/6 mice. Representative microphotographs of the pulmonary arteries in C57BL/6 mice and MRL/lpr mice stained by TUNEL staining (B). TUNEL-positive cells were indicated by red arrows. The graph showed the percentage of TUNEL-positive nuclei in medial smooth muscle layer (open bar; C57BL/6 mice, solid bar; MRL/lpr mice). Results are expressed as mean ± S.D. of 5 animals. *P<0.05 vs. C57BL/6 mice.

It has been reported that survivin plays an important role in pulmonary arterial smooth muscle cells proliferation and resistance against apoptosis [19, 20]. Therefore, we investigated survivin expression in the lung tissue of the MRL/lpr mice by western blotting. As shown in Fig 6A, survivin was upregulated in the lung tissue of the MRL/lpr mice. TUNEL staining revealed decreased TUNEL-positive nuclei in the medial smooth muscle layer of the MRL/lpr mice (22.6 ± 5.1 vs. 28.8 ± 2.9%, P < 0.05), (Fig 6B). These results suggested that increased survivin expression was one of the mechanisms of pulmonary arterial medial wall thickening in the MRL/lpr mice.

## Discussion

In the present study, we reported for the first time elevated RVSP, RV hypertrophy, medial wall thickening, and muscularization of pulmonary arteries in MRL/lpr mice. The phosphorylation disorder of eNOS, elevation of prepro-ET-1 and the upregulation of survivin were considered as the possible molecular mechanisms.

It has been reported that the serum levels of anti-dsDNA antibodies in MRL/lpr mice are already high at the age of 14 weeks, and further elevation of anti-dsDNA antibody, as well as the decline of C3 level, is also observed after 14 weeks [10]. Thus, the MRL/lpr mice used in the current study were at a relatively early stage of lupus; however, pulmonary vascular lesions were already advanced. In addition, we demonstrated that the deposition of IgG and C3 to pulmonary vessels was not directly related to the onset or progression of pulmonary vascular remodeling in the present study. Cytokine profile of the lung tissue of the MRL/lpr mice exhibited a shift of Th1/Th2 balance toward Th1 polarization as well as IL-17A elevation. However, it is still unclear how cytokine imbalance contributes to the onset of pulmonary hypertension in CTD. Mechanisms need to be elucidated further.

The pulmonary vessel morphology of the MRL/lpr mice in the current study presented almost the same findings as pulmonary arterial hypertension, which was characterized by medial wall thickening due to smooth muscle proliferation and the transition from non-muscular artery to muscular artery. However, the plexiform lesion, which is histopathologically the most advanced condition, was not observed. This result might be related to the fact that the degree of the RVSP elevation in the MRL/lpr mice was relatively low compared to other popular models, such as mice administered with monocrotaline, or VEGF inhibitor-hypoxia [8, 9]. In addition, left ventricular fibrosis, interstitial pneumonia, and pulmonary vein lesions, which are often found in CTD, especially scleroderma, were not observed in the MRL/lpr mice of the current study. These facts suggest that the MRL/lpr mouse was a disease model closer to SLE or Sjogren's syndrome, not scleroderma, not only in nephritis but also in pulmonary hypertension.

Fagan et al. reported that more than 50% of eNOS is required to maintain normal pulmonary vascular tone [16]. In the current study, when compared to the C57BL/6 mice, although the eNOS expression of the MRL/lpr mice did not differ significantly, eNOS phosphorylation was significantly lower. Since prepro-ET-1 is excessively produced in the lung tissue of the MRL/lpr mice and ET-1 has been reported to have an inhibitory effect on eNOS activation [21], decreased eNOS activation in MRL/lpr mice might be caused by ET-1 overexpression.

Although the role of survivin is important for pulmonary medial wall thickening in pulmonary hypertension [19, 22, 23], there have been no studies on the association between CTD-PH and survivin. We showed that survivin expression was upregulated as similar to other pulmonary hypertension models in the lung tissue of the MRL/lpr mice. In addition, since it has been reported that the expression of survivin is promoted by ET-1 [20], there is a possibility that a similar mechanism is involved in the lung tissue of MRL/lpr mice.

## Study limitations

There were some limitations in this study. 1 The effect of pulmonary hypertension on mouse survival was not detected since identification of the cause of death requires a long time and a large number of mice, and is difficult. 2 NO production in the pulmonary artery was not be measured due to the technical difficulty in isolation of endothelial cells or the pulmonary artery.

## Conclusion

**Fig 7:**
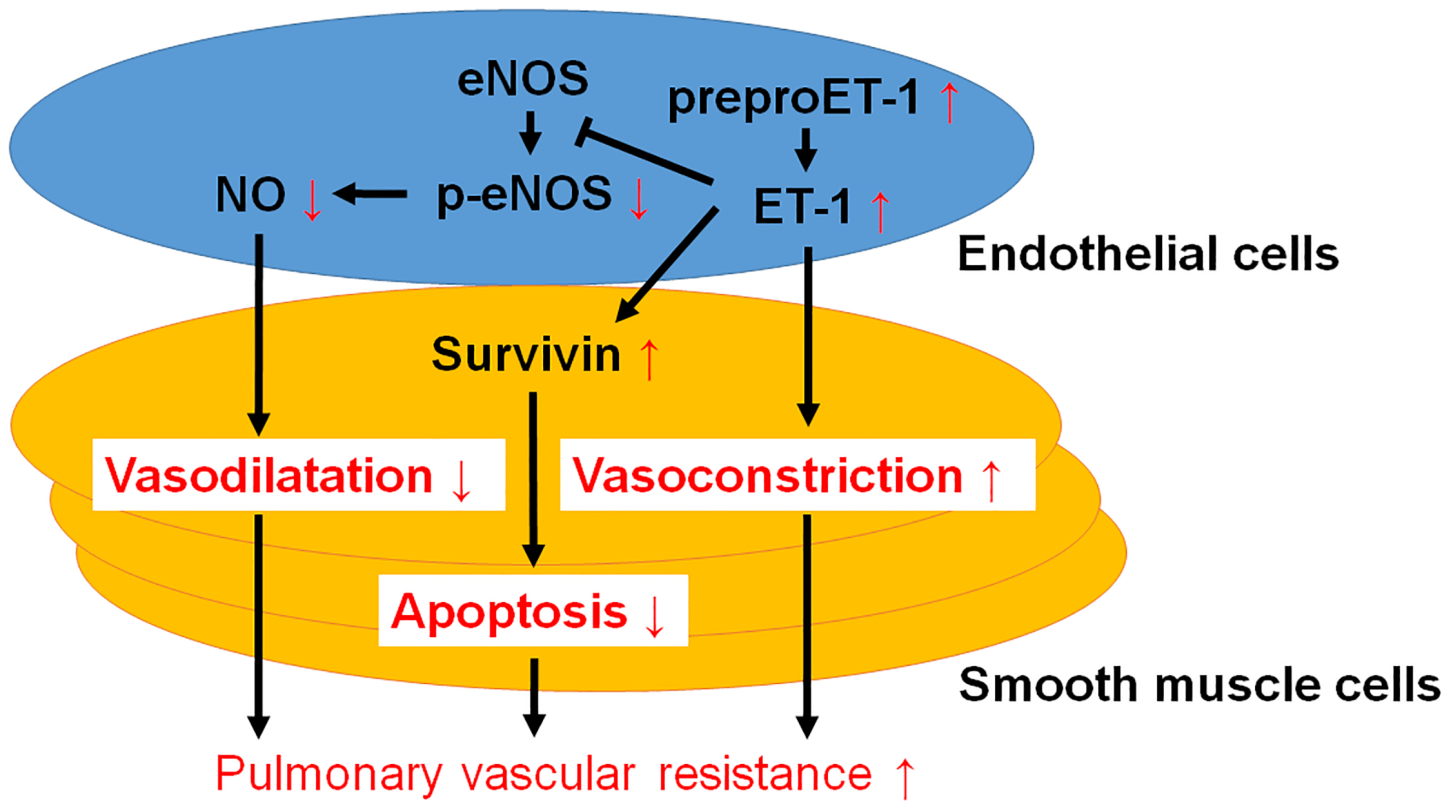
Schema of the signaling pathways in pulmonary artery endothelial cells and smooth muscle cells in MRL/lpr mice. Vasoconstriction predominates over vasodilatation due to NO impairment and upregulated prepro-ET-1 expression. Survivin promoted smooth muscle cells proliferation and resistance of apoptosis in MRL/lpr mice.

In the present study, we demonstrated for the first time that MRL/lpr mice spontaneously developed pulmonary arterial hypertension caused by an imbalance of vasodilation and vasoconstriction, as well as organic vessel stenosis. In addition, eNOS, ET-1 and survivin were found to play pivotal roles in the mechanism of pulmonary hypertension in MRL/lpr mice (Fig 7). Although further studies are required to elucidate the mechanisms of CTD-PH, MRL/lpr mice may be a useful model for the investigation of its pathophysiology.

## Supporting information

S1 File NC3Rs ARRIVE guidelines checklist. (PDF)
